# Exploring the combinatorial explosion of amine–acid reaction space via graph editing

**DOI:** 10.1038/s42004-024-01101-w

**Published:** 2024-02-03

**Authors:** Rui Zhang, Babak Mahjour, Andrew Outlaw, Andrew McGrath, Tim Hopper, Brian Kelley, W. Patrick Walters, Tim Cernak

**Affiliations:** 1 Department of Chemistry, University of Michigan, Ann Arbor, MI USA; 2 Department of Medicinal Chemistry, University of Michigan, Ann Arbor, MI USA; 3 Relay Therapeutics, Cambridge, MA USA

**Keywords:** Cheminformatics, Chemical libraries, Combinatorial libraries, Combinatorial libraries

## Abstract

Amines and carboxylic acids are abundant chemical feedstocks that are nearly exclusively united via the amide coupling reaction. The disproportionate use of the amide coupling leaves a large section of unexplored reaction space between amines and acids: two of the most common chemical building blocks. Herein we conduct a thorough exploration of amine–acid reaction space via systematic enumeration of reactions involving a simple amine–carboxylic acid pair. This approach to chemical space exploration investigates the coarse and fine modulation of physicochemical properties and molecular shapes. With the invention of reaction methods becoming increasingly automated and bringing conceptual reactions into reality, our map provides an entirely new axis of chemical space exploration for rational property design.

## Abstract (web summary)

Amines and carboxylic acids are abundant chemical feedstocks, however, the current reaction space of those two building blocks is focused on amide coupling. Here, the authors extensively explore the amine–acid reaction space via systematic reaction enumeration using graph editing and demonstrate its utility in retrosynthetic analysis as well as late-stage diversification.

## Introduction

**Fig. 1:**
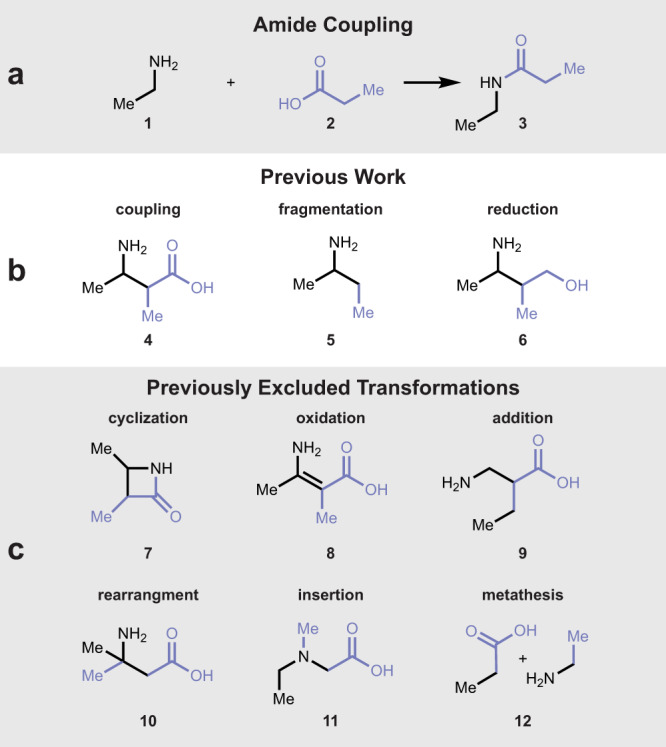
Venturing beyond the amide coupling for amine–carboxylic acid reactions. **a** Given an amine **1** and carboxylic acid **2**, the most popular transformation to unite this pair of building blocks produces the amide **3**. **b** Coupling products arising from a curated subset of chemical transformations charted by our prior work. **c** Examples of transformations excluded from our prior work.

Amines and carboxylic acids are two widely available functional groups that are classically united through the amide coupling reaction (*cf*. **1** + **2** → **3**, Fig. 1a), a tried-and-true chemistry that has become the most popular reaction for pharmaceutical explorations of chemical space^1^. Optimization of molecular properties is typically achieved through modification of starting materials or post-coupling functionalization. This pattern-following strategy of forming amide bonds from amines and acids ignores an untapped vector of chemical space exploration that employs diverse and conceivable chemical transformations in uniting these feedstock chemicals.

Our group has recently mapped amine–acid coupling reactions to explore the interplay of chemical transformations and physicochemical properties, as well as illuminate gaps in the synthetic chemistry toolbox (Fig. 1b)^2^. This initial study of the amine–acid coupling system, while agnostic of reaction mechanism, only focused on coupling reactions that seemed plausible. In brief, amines and acids were computationally united at the functional group atoms themselves (–NH_2_ / –CO_2_H), as well as at each partner’s α and β carbon atoms, utilizing a curated list of popular transformation modes such as coupling (**4**), fragmentation (**5**) and reduction (**6**). The virtual products generated span a wide range of physicochemical properties, suggesting that macroscopic properties such as solubility, cell permeability or metabolic stability could be influenced at the chemical transformation level. The structural motifs arising from these transformations were also found as substructures in many drugs, highlighting the potential value of these hypothetical transformations in synthetic and medicinal chemistry. However, the curated list of transformations focused on reaction plausibility instead of exhaustive enumeration, intentionally excluding many conceivable transformations where multiple bonds were formed or broken during the reaction. These include many popular transformations (Fig. 1c), such as cyclization (**7**), where multiple bonds are made between the amine–acid pair, oxidation of the bond forged during coupling (**8**), as well as less intuitive instances where at least one of the substrates is fragmented (e.g., addition (**9**), rearrangement (**10**), insertion (**11**) and metathesis (**12**)). We also excluded *sp*-hybridized atoms from our earlier model to focus on *sp*^2^- and *sp*^3^-hybrized atoms only.

A reaction enumeration study agnostic of reaction mechanism, or even conceived plausibility, would be an important advance. One way to achieve this is through exhaustive enumeration of molecular graphs. Graph theory concepts, wherein organic molecules are represented with atoms as nodes and bonds as edges, have been applied as early as 1875 to enumerate selected molecule classes, such as branched alkanes^3,4^, alkyl alcohols^5,6^ and cyclic carbon skeletons^7^. Such applications later evolved to systematically encode structures^8^ and reactions through matrices^9^, most notably in work by Ugi, Dugundji and coworkers^10–14^, that enabled computer-assisted discovery of pericyclic reactions^15,16^. These initial studies, while mathematically rigourous and theoretically effective, were hamstrung by contemporary scientific knowledge and hardware limitations. String-based reaction cataloging methods based on redox state changes, size of reaction center^17^ and identity of reactant atoms^18–22^, as well as fingerprint methods^23^ are also known. Reaction enumeration strategies built upon semantics must be constantly updated as new reactions and reaction types are continually discovered. On the other hand, combinatorial explosion resulting from enumeration methods can lead to large datasets that challenge the data handling capabilities of most computers.

Several modern reaction and structure enumeration methods build upon matrix encoding of molecules and transformations, applying them for molecular property and reaction product prediction^24,25^ or exploration of the transformation space of small molecules^26^. Graph-based structural enumeration has also been used to exhaustively enumerate chemical spaces bound by criteria such as ring count^27^ or atom count^28^. Other works employ generative algorithms to explore chemical space through various molecular representations, such as simplified molecular-input line-entry system^29^ (SMILES)^30,31^, molecular graphs^32^, DeepSMILES^33^, Self-Referencing Embedded Strings (SELFIES)^34^, and Sonic Architecture for Molecule Production and Live-Input Encoding Software (SAMPLES)^35^.

Among the modern ultra-large virtual libraries that have been reported, those that are generated through coupling of diverse building blocks employ only a small subset of curated reactions that are experimentally robust^24,25,36–44^. On the other hand, libraries that employ extensive structural enumeration focus on chemical space^28^—the *molecules* that can exist—but not transformation space, which encompasses the *reactions* that can exist.

Our goal is to complement these models with an extensive exploration of the transformation space spanned by reactions focused on a single substrate pair—namely an amine and a carboxylic acid. Importantly, we are agnostic of the experimental feasibility of each transformation at this stage. Since new reactivities are discovered every day, we anticipate that experimentally viable reaction conditions could be identified for especially desirable amine–acid transformations. While some of these proposed reactions may seem experimentally inconceivable today, the continual evolution of synthetic methods, coupled with modern high-throughput experimentation techniques^45–49^ brings even the most esoteric transformations closer to reality.

**Fig. 2:**
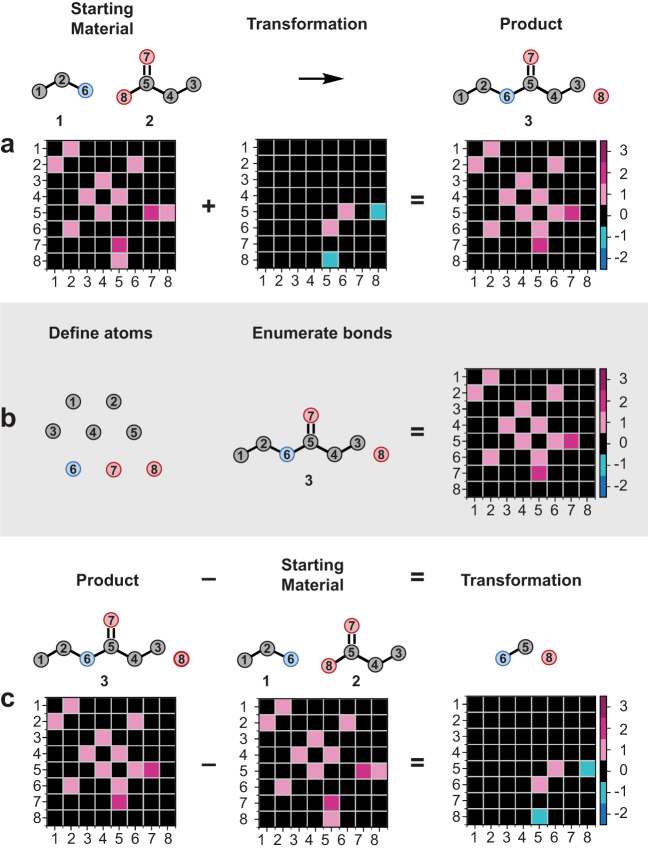
Mathematical basis for matrix-encoded molecular structures and reactions. **a** Demonstration of matrix-encoding of reaction from panel **1a**. In the molecular diagrams, white circles represent carbon, blue circles nitrogen, and red circles oxygen. The numbers in the adjacency matrix correspond to the atom indices in the cartoon atoms, while the color represents bond order. **b** Workflow for exhaustive generation of amine–acid coupling products. **c** The difference between the product matrix and starting material matrix is the transformation matrix.

The amine–acid pair was specifically chosen because of the ubiquity of these feedstock building blocks, the abundance of C, H, N, and O atom arrangements in drugs and natural products, and to contrast the dominance of the amide coupling as the most popular reaction in drug discovery^1^. Matrix variants have been developed to represent chemical transformations as well as molecular systems, such as bond/edge matrices^50,51^, generalized graph matrices^52,53^, 3D molecular graphs^54–56^, atom-bond connectivity matrices^57–61^, and other graph theoretical matrices^62,63^. In such applications, each row of the matrix corresponds to an atom, and at least half of the off-diagonal entries encode information about the bonds between all atom pairs in the system. When used to describe molecular systems, the off-diagonal entries encode bond orders, while in describing reactions, they encode changes in bond order. For this work, we used a matrix difference approach to exhaustively encode the reaction space of covering the NH_2_ and CO_2_H atoms, in addition to the α and β-carbon atoms of each amine or acid partner (Fig. 2a).

The upper bound to the number of products accessible by an amine–acid coupling is the total number of ways their constituent atoms, or a subset thereof, can be covalently attached without violating valency rules. Thereafter, the transformation from starting materials to products can be unambiguously described by listing the bonds broken and formed, which is obtained by taking the difference of the product and starting material matrices. Only neutral products were considered, although charged species, such as quaternary ammonium ions, are conceivable.

Figure 2a demonstrates a simple example with the coupling of ethylamine (**1**) and propanoic acid (**2**) to form amide **3**. First, a matrix is generated for the starting system. During this reaction, the bond order between N6 and C5 increases by one, while the bond order between C5 and O8 decreases by one. Addition of this transformation matrix to that of the starting materials then gives the amide product **3**. This same transformation matrix can be obtained by first separating this two-carbon amine and three-carbon carboxylic acid system into constituent atoms (Fig. 2b), then obtaining amide **3** by adding entries into a blank adjacency matrix. Along with **3**, the rest of the amine–acid coupling system was also exhaustively generated, constrained only by observing the octet rule, and allowing no formal charge on any single atom. In analogy to the subtraction method reported by Dugundji and Ugi^10^ as well as Schneider and colleagues^23^, the transformation matrices (Fig. 2c) were obtained by subtracting the adjacency matrix of the reactants from the adjacency matrix of the products.

Our matrix enumeration method demonstrated that expansion of the amine–acid coupling toolbox enables higher accessibility of chemical space, in terms of both physicochemical properties and shape. When the same set of transformations is applied towards coupling of larger amine and acid building blocks, a similar variation of properties was observed, although the coverage of some properties, such as shape, are attenuated. Compared to traditional virtual libraries generated by coupling diverse building blocks with a few established reactions, our complementary library, created through uniting a single building block pair with diverse reactions, performed comparably across three computational docking studies.

The structures generated through enumerating amine–carboxylic acid reactions can be used to screen compound libraries for retrosynthetic opportunities, and evaluate the significance of each amine–acid coupling reaction in synthesis of these libraries. Through exploring a drug structure database, several high-impact reactions have been identified and discovered by our group, in addition to a new coupling-rearrangement reaction that we will present herein.

## Results

### Physicochemical and shape space coverage

**Fig. 3:**
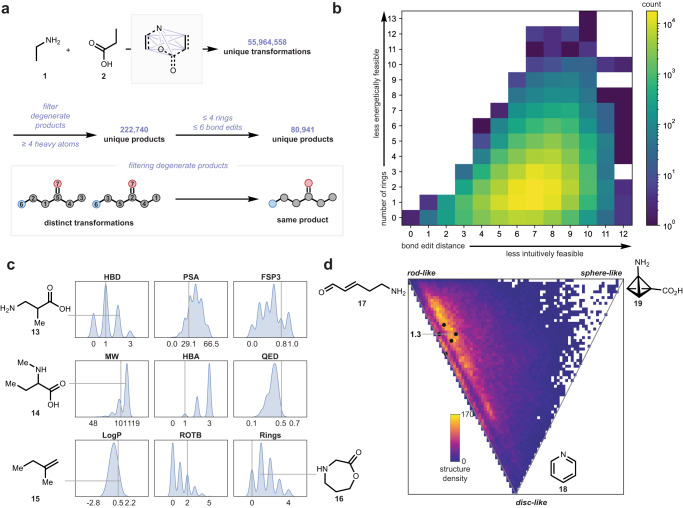
Process of virtual amine–carboxylic acid reaction enumeration and analysis of results. **a** Schematic of enumeration from amine **1** and acid **2** to yield ~56 million unique transformation matrices, which are filtered first to 222,740 unique products assuming carbon and oxygen atoms are degenerate, and further to 80,941 unique products after eliminating highly improbable structures. **b** Two-dimensional histogram showing distribution of ring count and bond edit distance of the initial 222,740 products. **c** Kernel density estimate (KDE) plots of various physiochemical properties of the expanded amine-acid coupling system, along with selected products. The respective property of the classic amide is shown by the vertical gray line. HBD hydrogen bond donor, PSA polar surface area, FSP3 fraction *sp*^*3*^-atoms, MW molecular weight, HBA hydrogen bond acceptors, QED quantitative estimate of drug-likedness, LogP partition coefficient, ROTB number of rotatable bonds, Rings number of rings. **d** Principal Moment of Inertia (PMI) ratio distributions of all products from the expanded enumeration.

This method generated a vast initial count of 55,964,558 conceivable transformation matrices (Fig. 3a). The quantity of viable transformations is remarkable since only eight atoms were considered. It is also significant that each of these 55 million reactions are treated as a single stereoisomer, and the space would expand considerably if all possible stereoisomers were considered. As well, the exclusion of atoms with a formal charge filtered out chemically reasonable functionalities like the nitro group or trialkylammonium salts, whose inclusion would further expand the space. Since the two oxygen atoms on a carboxylic acid are almost always chemically equivalent, the total number of products can be reduced by half to 23,829,176, unless isotopic labeling of ^16^O versus ^18^O is incorporated. In simple amine–acid systems, some carbon atoms may also be degenerate, resulting in two transformations producing the same product. For instance, considering the coupling of **1** and **2** with the loss of NH_2_ and CO_2_H, there are many conceivable ways to arrive at butane. If all five carbon atoms are considered degenerate, that is, with no α- or β-substitution or isotopic labeling, and only products with four or more heavy atoms are considered, the number of unique conceivable products is further reduced from 23,829,176 to 222,740 using the chemoinformatics package RDKit for molecule generation via adjacency matrix^64^. Within this vast structural space, many products contained motifs that are energetically improbable, or structurally distant from the simple amine–acid building blocks. To limit the inclusion of such improbable structures, we eliminated any structure with more than 4 rings, or requiring more than 6 bond edits from the amine–acid substrates (Fig. 3b). This brought the structure count to a final 80,941 products.

Figure 3c shows the kernel density estimate (KDE) plot of several molecular properties for the enumerated set of 80,941 amine–acid products. The vertical line in each panel highlights the property of the corresponding *sp*^3^–*sp*^3^ amide (**3**) for comparison. Expansion of the reaction space has a variety of effects on molecular properties compared to our earlier map^2^. While the amide product **3** has one hydrogen bond donor (HBD), the matrix enumeration set was allowed to break the carboxylic acid moiety into two alcohol groups, each being an HBD, accounting for the small peak at HBD = 3. Molecular weight (MW) and number of hydrogen bond acceptors (HBA) skewed larger when the reaction space was expanded. Both observations can be attributed to the increase in structures that incorporate all carbon, nitrogen, and oxygen atoms (**13**, **14**, **16**). Since the number of possible structures increases with the number of atoms involved, expanding the reaction set affords an increase in the number of relatively massive structures, accounting for the shift in distribution to larger MW. The distribution of MW also has a long tail towards structures with low mass, as fragmentation transformations result in products with fewer atoms than the starting materials. The same reasoning holds for the large peak at HBA = 3, since more product substructures can incorporate all three polar atoms, as in **14**. Other properties distribute more to lower values, such as logP, due to polar small molecules, and number of rotatable bonds (ROTB), due to highly rigid caged structures.

Next, the coverage of shape space by the 80,941 matrix-enumerated products was examined for shape diversity using a principal moment of inertia (PMI) ratio analysis^64^ (Fig. 3d). The enumerated reaction space covers an enormous diversity of three-dimensional shapes, including rod-like molecules such as **17**, disc-like molecules such as **18**, and sphere-like molecules such as **19**. The *sp*^3^–*sp*^3^ amide coupling product **3**, being a nearly linear molecule, sits near the upper left corner of the PMI plot. Upon application of matrix enumeration, the available molecular shapes cover a much larger portion of the PMI plot, suggesting that amines and acids can in principle unite to form a wide diversity of molecular shapes. Analogous plots of Fig. 3c, d were also produced for the full set of 222,740 structures and showed no significant expansion of property or shape space (Fig. S1), affirming that the transformation and energetic feasibility filters we applied are not significantly truncating the available chemical space.

### Application towards late-stage diversification

**Fig. 4:**
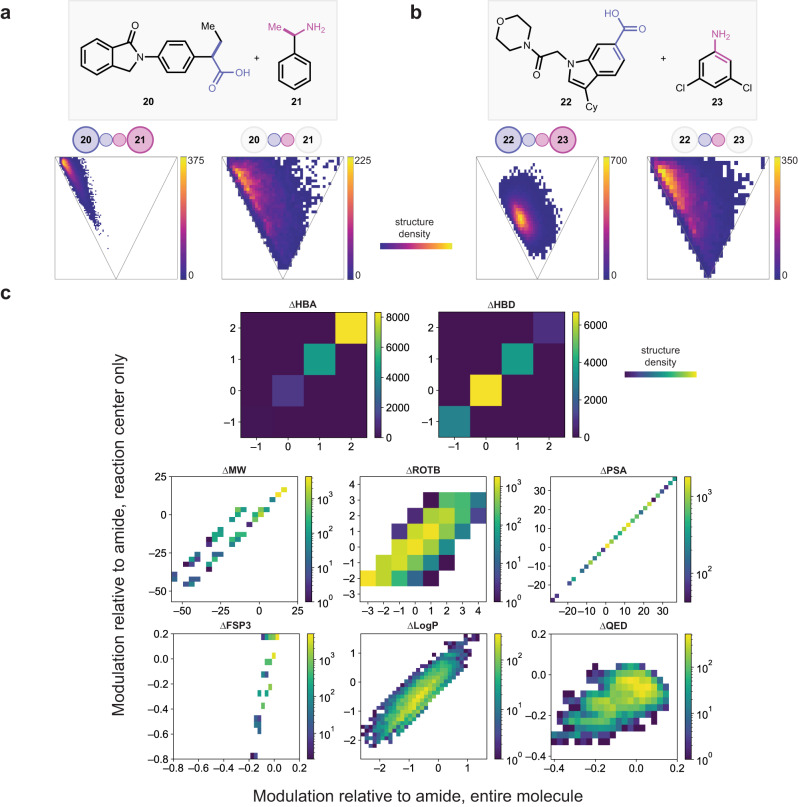
PMI ratio and joint distribution plots showing shape and property space distribution of reaction enumerated late-stage diversification of drug-like molecules. **a** An *sp*^3^–*sp*^3^ coupling followed by PMI ratio plots of products. The plots are labeled by the region that was analyzed. The smaller circles represent the reaction center, while the larger circle represents all other atoms. If all circles in the label are shaded, then the entire molecule’s shape was computed. If only the smaller circles are shaded, then only atoms at the reaction center were analyzed (cf. Fig. 3d). **b** An *sp*^2^–*sp*^2^ coupling, analyzed with the same method as part **a**. **c** Joint distribution plots of physicochemical property modulation within the system depicted in part **a** (**20** + **21**). See Fig. S2 in Supporting Information for corresponding plots for **22** + **23**. The *x*-axes show modulation of the entire molecule relative to the amide, while the y-axes show modulation of only the atoms at the reaction center. HBD hydrogen bond donor, PSA polar surface area, FSP3 fraction *sp*^3^-atoms, MW molecular weight, HBA hydrogen bond acceptors, QED quantitative estimate of drug-likeness, LogP partition coefficient, ROTB number of rotatable bonds, Rings number of rings.

To explore our hypothesis that property modulation can be achieved by varying transformation, as a complement to varying building blocks, we applied matrix-derived amine–acid transformation enumeration as a strategy towards the virtual late-stage diversification of pharmaceuticals. Traditionally, this has been achieved through the selection of alternative building blocks^65–68^, which are then united with the substrate through popular reactions such as the amide coupling, Suzuki coupling and Buchwald-Hartwig coupling. As a complement to this method, we applied matrix-derived amine–acid transformations to two druglike molecules, one containing a *sp*^3^-acid (**20**) (Fig. 4a) and one containing an *sp*^2^-acid (**22**) (Fig. 4b), with a simple amine of the corresponding hybridization (**21** and **23**). This time, to keep the products aligned with desired outcomes of late-stage diversification, we removed products with high ring strain from both product sets, removed products that lost a significant substituent in **20** and **21**, and retained a six-membered ring at the reacting atoms in **22** and **23**. To compare the diversification of physicochemical properties brought about by the reaction center to that of the substituents, all matrices that gave valid products while coupling **20** and **21** were extracted, and used to transform the simple amine–acid pair **1** and **2**, generating a complementary set of only the reaction center, represented by the smaller colored circles in Fig. 4a, b.

As illustrated in Fig. 4, there is considerable variation in molecular shape (Fig. 4a, b) and physicochemical properties (Fig. 4c). The influence of the substrate is prominent in the modulation of molecular shape. Though the reacting atoms of the carboxylic acid and amine functional groups span a more diverse shape space, the starting materials are larger (*cf*. **20**/**21** and **22**/**23** versus **1**/**2**) so they exert more influence on the final product’s shape. Between **20** and **21**, the aggregate PMI ratios skew towards the 1D-2D line but still distribute towards the 3D region of the PMI plot due to the relatively smaller substituent size compared to **22** and **23**. The products in the latter set were also filtered to those retaining the aromaticity of the benzoic acid and aniline moieties, so there are fewer molecules and less coverage in this *sp*^2^–*sp*^2^ pairing. Considering physicochemical properties (Fig. 4c, only properties for the pairing of **20** and **21** are shown, see Fig. S2 in Supporting Information for complementary plots for **22** and **23**), where the modulation of each property in regard to the amide is plotted on the x-axis for the whole molecule, and y-axis for the reaction center, HBA and HBD vary in similar amounts, since each datapoint represents the same transformation, as HBA and HBD are context-independent and additive properties. The parallel diagonal bands in MW, and some extent LogP reflect a divergence between transformations that lose atoms carrying functional groups (the methyl groups on the α-carbons of **20** and **21**), while movement along the bands reflect simultaneous transformations that occur on the other atoms (such as the acid oxygens). Meanwhile, ROTB exhibits a broader spread, as it is influenced by structural motifs on both the reacting atoms and the other atoms of the building blocks. The change in fraction *sp*−3 atoms (FSP3) varies more discretely for the reaction center, since there are only 8 atoms in the model system, hence the fraction can only change in multiples of 0.125. Lastly, quantitative estimate of drug-likeness (QED), being an aggregate property, shows no clear trend, as evidenced by differences in the distribution of each property whether the full product or only the reacting atoms are considered.

### Virtual docking of compound libraries generated through diverse amine–acid coupling reactions

**Fig. 5:**
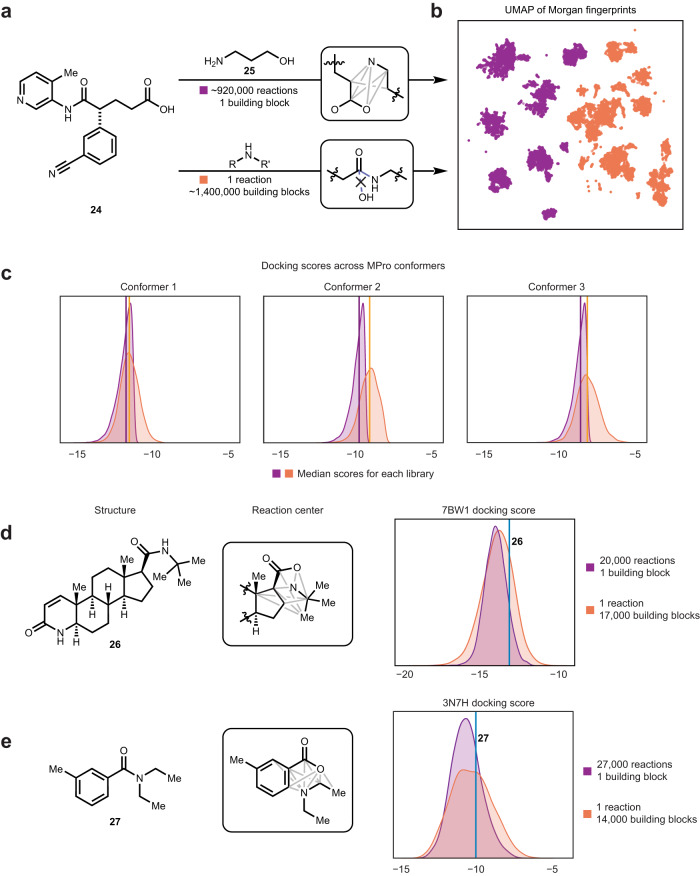
Generating large virtual libraries through application of diverse coupling reactions. **a** Workflow in generating two orthogonal virtual libraries. **b** Two-dimensional UMAP projection of the combined chemical space, using 1,024-bit Morgan fingerprints, colored by the library in which each molecule belongs in. **c** Docking energies of both libraries across three M^pro^ conformers. **d** Same workflow as 5a applied to finasteride (**26**), a schematic representation at the atoms at which transformations were permitted to occur, and distribution of docking scores. Filters were applied to include only products that preserved the local ring structure. **e** Same workflow as 5a applied to DEET (**27**).

To evaluate the performance of enumeration-derived virtual libraries in practice, an amine–acid pair (**24** and **25**) resembling known inhibitors of the SARS-CoV-2 main protease (M^pro^) was designed (Fig. 5a). Two orthogonal virtual libraries were created, one from enumerating amine–acid transformations between the pair, and the other from performing only amide coupling of **24** with diverse primary and secondary amines retrieved from the PubChem database^69^, filtered to less than 13 heavy atoms, and less than two total nitrogen and oxygen atoms, to maintain similar polarity between the two libraries.

Two-dimensional Uniform Manifold Approximation and Projection (UMAP)^70^ performed on 1,024-bit Morgan fingerprints^23^ of sampled structures in both libraries showed complementary coverage of chemical space with little overlap, illustrating that invention of diverse amine–acid reactions will allow access of chemical space previously untapped by modifying building blocks alone. Docking of both virtual libraries was performed across three M^pro^ conformers (Fig. 5c). Across all conformers, the library generated via diverse reactions consistently achieved docking energies with a narrower spread than the library from diverse amines, while also performing more favorably.

This study was repeated with two additional molecules— finasteride (Fig. 5d, **26**) docking with Steroid 5-alpha-reductase (7BW1)^71^, and diethyltoluamide (DEET) (Fig. 5e, **27**) with Odorant Binding Protein 1 (3N7H)^72^. Both small molecules were disconnected at their amide bond, and a virtual library generated through recombining the resultant amine–acid pair with diverse transformations. As both acid building blocks possess ring systems, the transformation products were filtered to structures that preserved these rings as well as aromaticity, when relevant (Fig. S3).

Compared to the M^pro^ inhibitor study, the library generated by combining one building block pair with diverse transformations did not produce a significantly different docking score distribution, but maintained a narrower spread. Interestingly, both methods of virtual library generation produced better average scores than the original amides, as indicated by the vertical lines in Fig. 5d, e. These studies reinforce our hypothesis that conducting diverse reactions between two building blocks allows for finer tuning of physicochemical properties than the traditional method of varying the building block itself. A future approach to in silico screening may be to first determine the target compound’s constituent building blocks, followed by probing the pair’s coupling space to determine the most favorable reaction to unite them with.

### Application towards retrosynthetic analysis

**Fig. 6:**
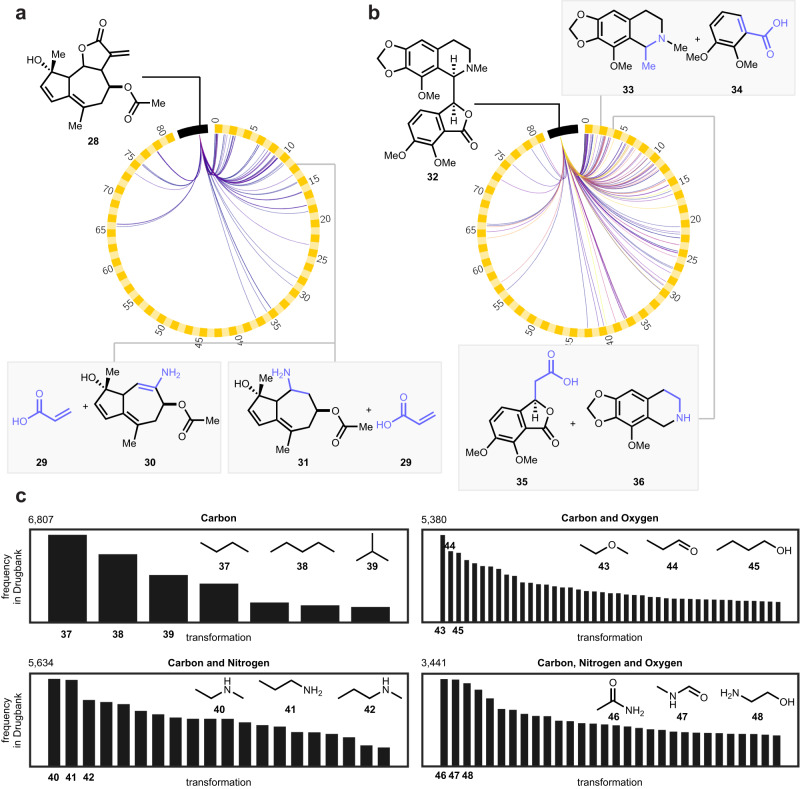
Using database searches to determine important reactions for discovery. Chord diagram showcasing connectivity of two drugs to enumerated amine–acid coupling products. Drug targets lie on the black band, and the exhaustive matrix enumeration products lie on the checkered yellow band. The color of the chord represents the substructure’s frequency of occurrence, ranging from one to greater than ten. **a** Two disconnections are shown for athamontanolide (**28**), both into acrylic acid (**29**) and an amine (**30, 31**) with new disconnections generated by the current enumeration workflow. **b** Similarly, noscapine (**32**) can be disconnected to amine **33** and acid **34**, or to alkyl acid **35** and secondary amine **36**. **c** The frequencies of the top 100 most abundant substructure matches in DrugBank are plotted as a histogram, categorized by their elemental makeup. The top three most abundant structures for each group are shown (**37**–**48**).

Since our strategy is to focus on reactions, the dataset of amine–acid transformations can also be used as a retrosynthetic strategy to disconnect complex molecules for total synthesis. We performed substructure searches of our enumerated products within the DrugBank^73^ database to inform potential retrosynthetic disconnections for synthetic planning of complex target molecules. Two natural products, athamontanolide (**28**) and noscapine (**32**), were selected for further analysis. The results of a search of the 80,941 enumerated product substructures in these two molecules are visualized as a chord diagram (Fig. 6), and detailed in Figures S4 and S5 for the two respective natural products. The high degree of connectivity demonstrates the many opportunities for retrosynthetic simplification that arise through the invention of new reactions.

The top 100 most frequently occurring matrix enumerated reaction product substructure matches in DrugBank are also displayed in Fig. 6c to highlight potentially impactful amine–acid reactions for development, and visualized in Fig. S6. The most abundant substructure containing only carbon is the C–C–C–C motif **37**. Meanwhile, the *sp*^*3*^–*sp*^*3*^ C–O coupling motif **43** and *sp*^*3*^–*sp*^*3*^ C–N coupling **40** are the most abundant in structures containing only carbon and oxygen, and carbon and nitrogen respectively. Together, these findings suggest that these three transformations could be valuable in drug discovery and natural product synthesis, among which we have recently reported C–C coupling^74,75^ and C–O coupling^76,77^. The most abundant substructures with C, N and O atoms contain fragments of the amide bond (**46,** **47**), which once more underscores the prevalence of the amide coupling in drug discovery, but also opportunities in accessing complementary chemical space with amine–acid couplings that do not produce the amide, yet preserve C, N and O atoms. A wider substructure search within Drugbank was performed with all 222,740 amine–acid coupling products, and the results are visualized in Fig. S7.

### Experimental demonstration of reaction space expansion

**Fig. 7:**
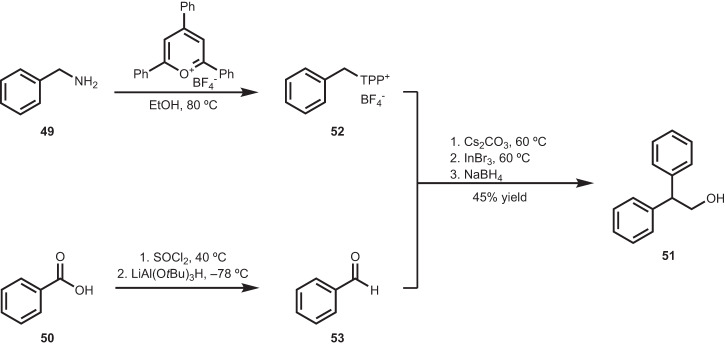
Experimental expansion of the amine–acid coupling space through development of reactivities beyond the amide coupling. An amine–acid coupling-rearrangement reaction results in an α,α-disubstituted alcohol.

To affirm the experimental viability of our theoretical work, our lab is seeking out reaction conditions leading to unconventional amine–acid couplings^74^. We have previously reported *sp*^2^ amine–acid coupling^76^, *sp*^3^–*sp*^3^ C–C coupling^78^, *sp*^3^–*sp*^3^ C–O coupling^77^, and *sp*^3^–*sp*^2^ C–C coupling^75^, and here demonstrate an additional transformation, where activated benzylamine (**49**) and benzoic acid (**50**) couple to form alcohol **51** (Fig. 7)﻿.** 49** is activated as the benzyl Katritzky salt (**52**), while **50** is reduced to the corresponding benzaldehyde (**53**). The pair of building blocks couple and subsequently afford **51** through migration of the acid α- and β- carbons.

## Discussion

Applying the graph editing approach described herein to amine–acid coupling reactions, more than 55 million potential transformations emerged when no filters for atom degeneracy were applied. This is remarkable and opens the gates to a wide range of chemical space. Due to the popularity of the amide coupling, many institutions have abundant amounts of amine and acid building blocks in hand. Each amine–acid coupling identified and discovered represents an additional method to couple these building blocks, accessing novel properties and structures.

While there is precedence for the synthesis of some structures presented in this manuscript from functional groups other than amines and carboxylic acids, we do not believe that this obviates the value of discovering amine–acid couplings that arrive at the same motifs. Among commercially available building blocks, each reagent class (amines, bromides, boronates, etc.) possesses its own unique coverage of chemical diversity. Hence, discovery of numerous complementary methods to unite various combinations of functional groups will lead to access of these complementary chemical spaces from building blocks currently available on the market.

Our reaction enumeration approach also provides a new axis for ultra-large virtual library generation. Ultra-large virtual libraries have seen a surge in interest recently^37–44^, due to improvements in computing power. While the traditional approach necessarily gravitates to experimentally robust chemical reactions, it is becoming increasingly possible to automate the invention of new reaction methods. For this reason, it is critical to develop an understanding of which reactions can exist and the role they play in modulating physicochemical properties as detailed herein.

## Methods

All computation for the manuscript was performed in a Conda environment with the following packages: ipython 7.16.1, jupyterlab 3.1.4, matplotlib 3.3.4, numpy 1.19.2, pandas 1.1.3, RDKit 2019.09.3^79^, seaborn 0.11.1, tqdm 4.56.0, umap-learn 0.5.1, circos 0.69-9. All packages except Circos are installed via conda-forge or pip. Chord diagrams were plotted using the Circos package, downloaded via http://circos.ca/software/download/circos/.

### General Procedure 1: Interconverting between a matrix and its corresponding molecular system

To convert a molecular system to a matrix, the molecule(s) to be encoded are visualized, and all non-hydrogen atoms are assigned indices. The assignment of the indices does not affect the result, but the matrices will look more intuitive if adjoining atoms in the molecule are given indices that put them next to, or close to each other in the matrix. Alternate assignments can be selected to facilitate analysis, as long as these assignments do not change throughout creation and conversion of the matrices.

Next, each bond between heavy atoms is encoded as an integer, in the position corresponding to its neighboring atoms’ indices. In the examples shown in Fig. S8, since atoms #2 and #6 have a single bond between them, then the matrix entries (2,6) and (6,2) are set to 1. Similarly, the (5,7) and (7,5) entries are set to 2. Ionic and multi-center bonds are beyond the scope of this work, and interested readers are referred to the work of Ugi et al. on discussions of how these bonds can be encoded^12^.

To quickly encode larger systems, RDKit contains a built-in functionality to generate an adjacency matrix directly from mol objects, but one should note that the atom numbering will follow that of the mol object. Should the object be generated from a SMILES string, the atoms will be numbered by their order of appearance in the string. This may cause atoms close to each other to be far apart in the matrix, especially for branched and ring structures (Fig. S9). Note that a single matrix can encode multiple molecules, hence the choice by past scientists to use flexible terminology, such as systems and ensembles^10,12,13^, to describe the structures encoded.

To ensure consistent interconversion, the atomic numbers of the constituent atoms, in the order they appear in the adjacency matrix, should be stored alongside the matrix, since this information is not contained within the matrix. This process can now be reversed, to obtain a molecular system from its corresponding adjacency matrix.

First, a RWMol object is initialized. This is similar to RDKit’s regular mol object, but allows for atom and bond editing. The molecular system is then constructed by first iterating through the atomic numbers to place all individual atoms, then through all non-zero elements of the lower triangle of the adjacency matrix to place all bonds.

### General Procedure 2: Full molecular structure enumeration

To obtain all desired transformations for a molecular system, our approach is to first generate the set of all product structures that adhere to our boundary conditions (no violation of octet rule, no formal charge on any atom).

The procedure for generating these matrices are as follows:

1. Determine bonding parameters for all atoms. Every atom has two parameters: the maximum total bond order, *t*, originating from it, and the highest order for an individual bond, *b*. For example, a neutral nitrogen atom has (*t,b*) = (3,3), while a neutral carbon has (*t,b*) = (4,3) since, while carbon can have a total bond order of 4, it is not allowed to make quadruple bonds.
2. Determine the identity of the first row’s corresponding atom. Generate all possible rows that sum to *t* or below (since bonds to hydrogen are implied), with each entry having a maximum value of *b*, and the first entry being 0 (since an atom cannot bond with itself).
3. Starting with the first iteration of the first row, copy its second element into the first entry of the second row, since the matrix is symmetrical about the diagonal, and set the second entry of the second row to 0.
4. Using the second row atom’s values of *t* and *b*, generate all possible remaining variants of the second row.
5. Repeat steps 3 and 4 to generate subsequent rows, where the *n*th row is initialized by vertically stacking the previously generated rows into a rectangular matrix, copying the *n*th column into the *n*th row, then generating all permitted permutations of the *n+1*th element and beyond which the *t* and *b* values permit.
6. The last row does not need any generation, since it is a copy of the current matrix’s last column, with an appended 0.
7. For our work, matrices were converted into numpy 8-bit integer arrays to reduce memory usage. The matrices can be further compressed into sparse triangular matrices if desired.

Code for matrix generation is stored in 000_generate_matrices.ipynb, which calls 000_script.py.

### General Procedure 3: Generation of reaction matrices

Four initial systems were encoded, one for each permutation of hybridization of the amine and acid. These are visualized in Fig. S8 and stored in sm_amats.py.

For each product matrix generated in General Procedure 2, four sets of transformation matrices were obtained, each via subtracting one of the matrices in Fig. S8 from each product matrix. Bond edit distance for each transformation was obtained by first taking the absolute value of all transformation matrix elements, summing all of them up, and then dividing the result by 2 due to symmetry.

Code for computing bond edit distance is stored in 001_processing.ipynb in the compile_smiles_dists function. The notebook further computes, for each product structure, the number of heavy atoms and minimal bond edit distance across all four permutations of acid and amine hybridization. This data is saved as spreadsheet smiles_min_dist_natoms.csv.

### General Procedure 4: Drugbank substructure search and plotting

All desired molecules and enumerated substructures are loaded into RDKit as mol objects and sanitized. For each substructure, the frequency it occurs in the drug structure is computed and recorded. In plotting the chord diagram, the substructures are arrayed clockwise by increasing bond edit distance from the amine*–*acid starting materials. The more bonds that must be changed to convert the starting materials into the product, the higher is the reaction label number displayed around the periphery of the circle (Fig. S10). It should be noted that the number of bond edits from a given substrate pair does not necessarily correlate with synthetic ease. For example, the Diels-Alder reaction features six bond edits in the transformation matrix but experimentally occurs as a single concerted mechanistic step. In contrast a direct C–N bond cross-coupling transformation has only a single bond edit but experimentally requires several elementary steps in the form of a catalytic cycle including oxidative addition, reductive elimination and deprotonation^80^.

For this exercise, we chose to preserve the aromaticity of the enumerated and drug structures. When a molecule is loaded into RDKit, the user can choose to encode aromatic systems as conjugated single and double bonds^76^. While we initially chose to remove aromaticity markers, we found a high proportion of substructure matches to lie along aromatic rings, which point to a disconnection fragmenting those rings. To remove these energetically undesirable matches, aromaticity of all molecular structures was subsequently preserved (Fig. S11).

Code for substructure search is contained in 002_substructure_search.ipynb. The notebook takes as input smiles_min_dist_natoms.csv, appends the search result as an additional column in the spreadsheet, and saves the output as smiles_mindist_dbank.csv.

### General Procedure 5: Computation of principal moment of inertia (PMI) ratios

The concept of utilizing PMI ratios to visualize molecular shape space distribution was first published by Sauer and Schwarz^65^. The three PMIs of a molecule are computed and sorted in ascending order, then each of the first two PMIs are divided by the largest one to obtain normalized PMI ratios (NPRs), where NPR1 ≤ NPR2. NPR1 is then plotted against NPR2 to obtain the triangular plots.

In our work, each structure is converted by the RDKit package to a mol object, all hydrogens are added, and the molecule embedded in 3D with the ETKDG method developed by Riniker and Landrum^81^. The values of NPR1 and NPR2 are subsequently computed by built-in functions from the same package.

Code for computing PMI ratios and other physicochemical properties is stored in 003_smiles_basic_analysis.ipynb. The spreadsheet created from General Procedure 3 is read as input, physicochemical properties computed with built-in RDKit functionality, and results saved in smiles_min_dist_dbank_props.csv. PMI ratios for all structures are saved in smiles_pmi.csv. Note that a None result is produced if the RDKit fails to embed the structure, which is an occurrence in highly strained systems.

### General Procedure 6: Late-stage diversification

To conduct a molecular transformation using reaction matrices, the hybridization of the substrate amine and acid groups are noted, and the appropriate set of reaction matrices retrieved. Using RDKit, the pair of substrates is encoded as a single editable molecule. A substructure search is then performed in RDKit to identify and index the atoms at which the transformation will occur. Based on the encoding of the substrates, a mapping of atom index to matrix row is constructed (Fig. S12). Bond order changes are subsequently performed as per directed by the reaction matrix. Since the reaction matrices are generated assuming no substitution at the α and β carbons, this will lead to diversification products that violate the octet rule. These products are automatically filtered out through RDKit’s sanitization capability (Fig. S13, steps 1-4). Lastly, the molecules are passed through a structural filter that removes product with anti-Bredt and other strained ring motifs (Fig. S14). The *sp*^*2*^*–sp*^*2*^ coupling set is further filtered to remove products that have aromaticity of the substrates destroyed.

### General Procedure 7: Generation of corresponding reaction centers

All matrices that result in a valid molecule in the previous section have their indices noted. These matrices are subsequently retrieved and applied to the simple amine–acid system to generate the set of reaction centers, each corresponding to a functionalized drug molecule (Fig. S13, steps 5–6). Since the α and β carbons in their corresponding substrate are not degenerate, the set of reaction center structures was not further made unique.

Code for this section is stored in the late_stage_div folder. The notebooks for workflow depicted in Fig. S13 begin with 011 and 012 for the *sp*^3^ system, and 013 and 014 for the *sp*^2^ system, and output one spreadsheet each containing SMILES of enumerated products. PMI ratios are computed by notebooks prefixed with pmi, followed by the hybridization of the system.

### General Procedure 8: Generation, docking, and processing of complementary virtual libraries

Virtual reaction enumeration libraries of druglike molecules were generated via the workflow in General Procedure 6. An amine library was generated by first retrieving all structures in the PubChem database, filtering first to amines that do not contain any elements outside of H, B, C, N, O, F, P, S, Cl, Br and I, and then to amines that have less than 14 heavy atoms. Virtual amide libraries were then generated by coupling each amine to the acid drug building block. In the libraries for finasteride and DEET, amines were further filtered to those containing less than three N and O atoms combined.

The Openeye Toolkit 2021.2.0 was used for the docking protocols. Conformations were generated using OMEGA with default torsion libraries after selecting a reasonable tautomer and then performing stereo expansion for undefined stereo centers. The receptors for each protein were docked using FRED with default parameters.

After docking scores were obtained, filters were further applied to each library. All libraries generated through amine–acid reaction enumeration were filtered to six or less bond edits from the starting building blocks. The finasteride and DEET-based libraries generated through amide coupling were filtered to a maximum heavy atom count equal to that of the reaction enumeration library. This is 29 heavy atoms for finasteride and 15 for DEET. Docking score distributions for the M^Pro^ inhibitor analog filtered to the same rule (29 heavy atoms) are shown in Fig. S15. Trellised KDE plots for docking score against maximum heavy atom count are included as Figs. S16–S18.

Experimental details on the synthesis of **51, 52** and **53**, and NMR spectra of **51** (Fig. S19) can be found in the Supporting Information. Additional notes and workflows for selected General Procedures can be found in the Supplementary Notes.

### Supplementary information

Supporting Information

## Data Availability

All data visualized in the manuscript is generated from code in the public repository presented below. Drug structures were accessed from the Drugbank database via https://go.drugbank.com/releases, version 5.1.8.

## Code availability

All codes for matrix generation, molecular property computation, and figure plotting are available at https://github.com/cernaklab/acid-amine-enumeration-2. A demo of the same script be found at https://github.com/cernaklab/acid-amine-enumeration-2/tree/main/Demo. This folder contains the same hierarchy of script and directory paths as the main code, with only a few modifications to run on a smaller set of matrices and only utilizing one core.
