# Supplementary material for: Exploring the combinatorial explosion of amine–acid reaction space via graph editing
Source: Commun Chem. 2024 Feb 3;7:22. doi: 10.1038/s42004-024-01101-w (PMC10838272; doi:10.1038/s42004-024-01101-w)
Supplement: Supplementary file 1 — Supporting Information [file 42004_2024_1101_MOESM1_ESM.pdf]

Supporting Information for

Exploring the Combinatorial Explosion of Amine–Acid Reaction Space via Graph Editing

Rui Zhang,[1] Babak Mahjour,[2] Andrew Outlaw,[2] Andrew McGrath,[2] Tim Hopper,[3] Brian Kelley,[3] Pat Walters,[3] Tim Cernak[1,2]*

1. Department of Chemistry, University of Michigan, Ann Arbor, MI, USA
2. Department of Medicinal Chemistry, University of Michigan, Ann Arbor, MI, USA
3. Relay Therapeutics, Cambridge, MA, USA

Table of Contents

Figure S1. 3
Figure S2. 4
Figure S3 5
Figure S4 6
Figure S5 7
Figure S6 8
Figure S7 9
Figure S8 10
Figure S9 10
Figure S10 11
Figure S11 11
Figure S12 12
Figure S13 13
Figure S14 13
Figure S15. 14
Figure S16 15
Figure S17 15
Figure S18 16
Supplementary Notes.......... 17
Additional notes and/or workflow for selected General Procedures 17

General Procedure 1 17
General Procedure 2 18
General Procedure 3 18
General Procedure 4 19
General Procedure 5 19
General Procedure 6 20
General Procedure 7 21
Experimental details on the synthesis of **51**, **52** and **53**..21
Figure S19. 23

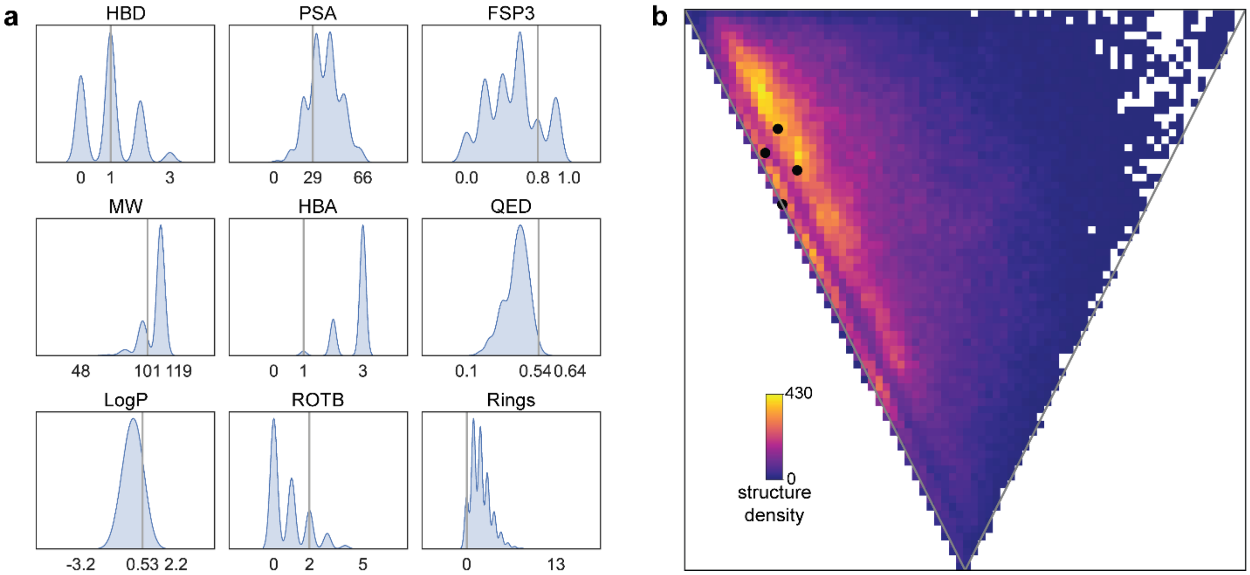

Figure S1. a. Kernel density estimate (KDE) plots of physicochemical properties of the full enumerated amine–acid coupling product set, comprising of 222,740 structures. **b.** Principal Moment of Inertia (PMI) ratio plots of the enumerated product set. The four black dots indicate the location of the four possible amide products.

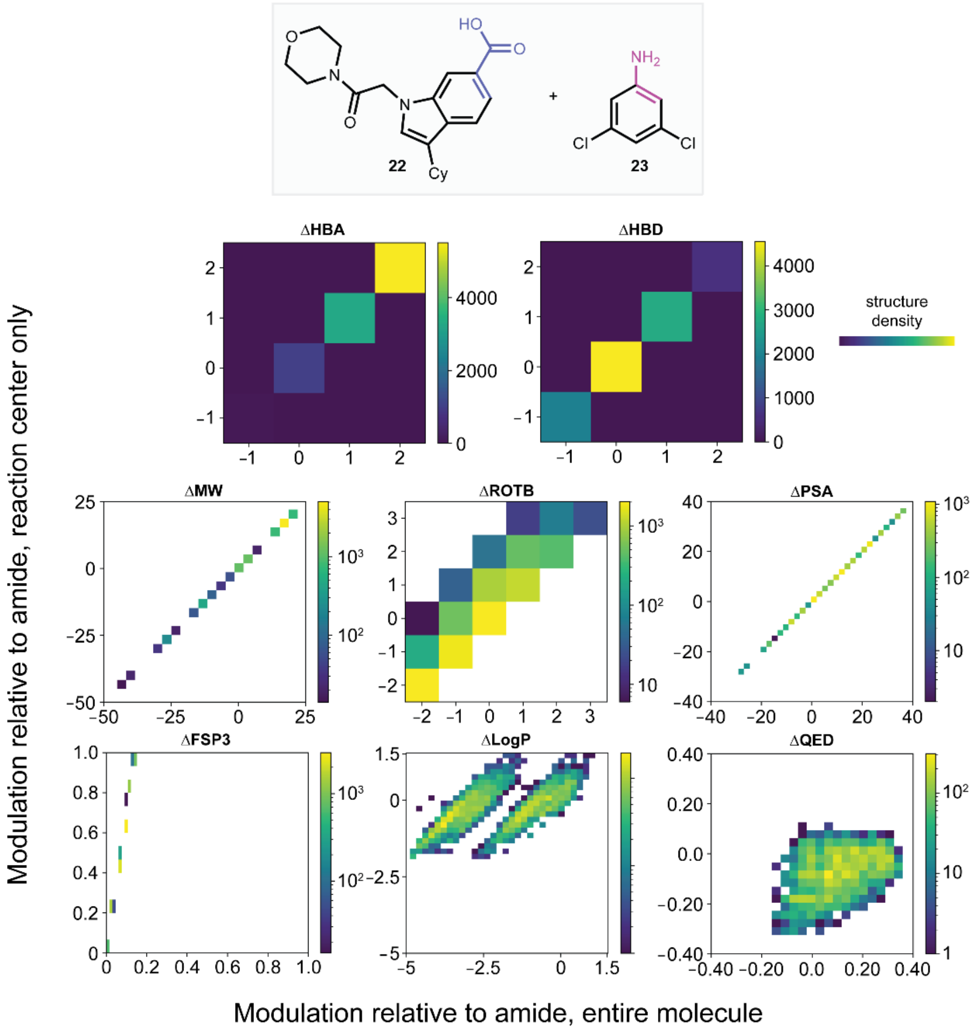

Figure S2. Joint distribution plots for late-stage diversification of molecule **22** with amine **23**.

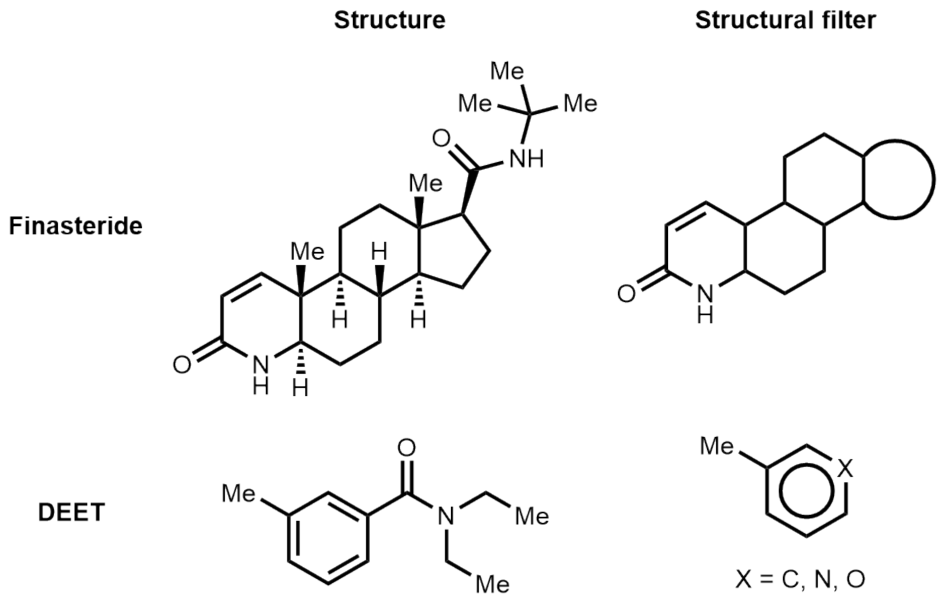

Figure S3. Structural filters applied to virtual libraries of finasteride and DEET.

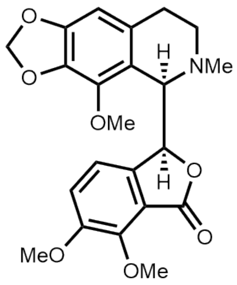

33

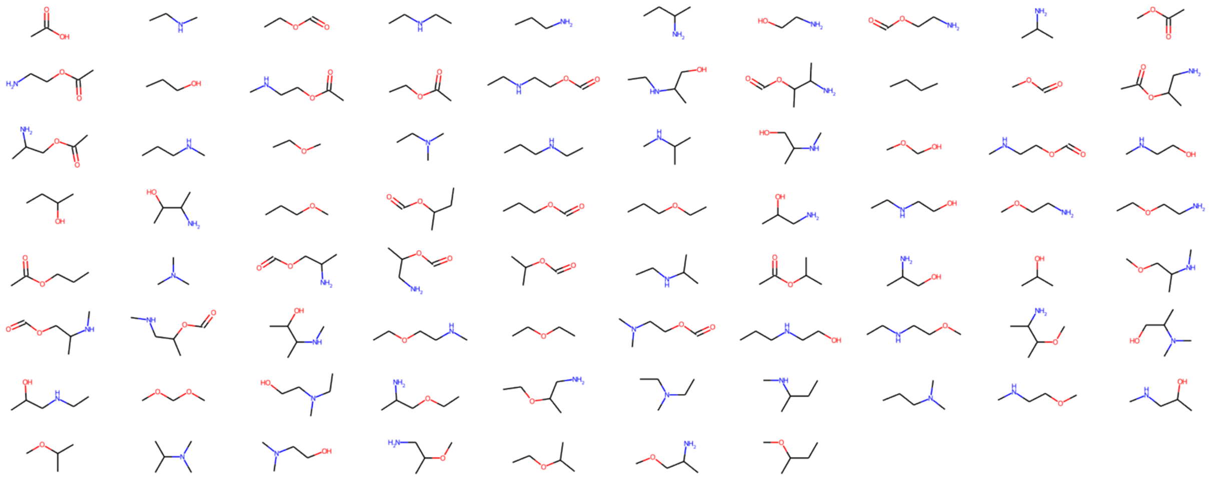

Figure S4. All amine–acid reaction products in this work found in noscapine. The structures are arrayed in increasing minimum graph edit distance from a simple 2-carbon amine and 3-carbon acid pair.

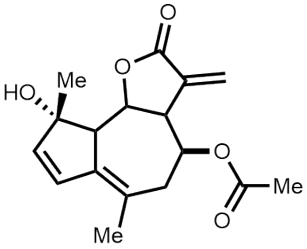

29

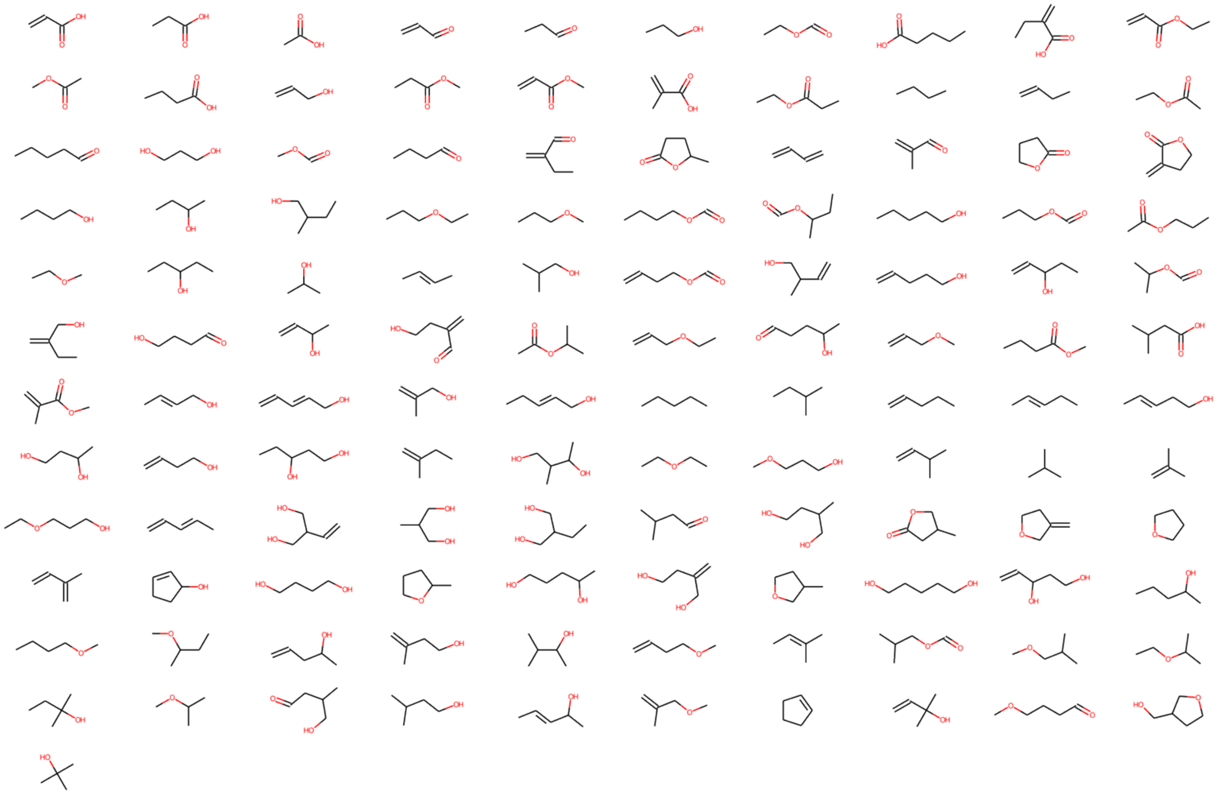

Figure S5. All amine–acid reaction products in this work found in athamontanolide. The structures are arrayed in increasing minimum graph edit distance from a simple 2-carbon amine and 3-carbon amine pair.

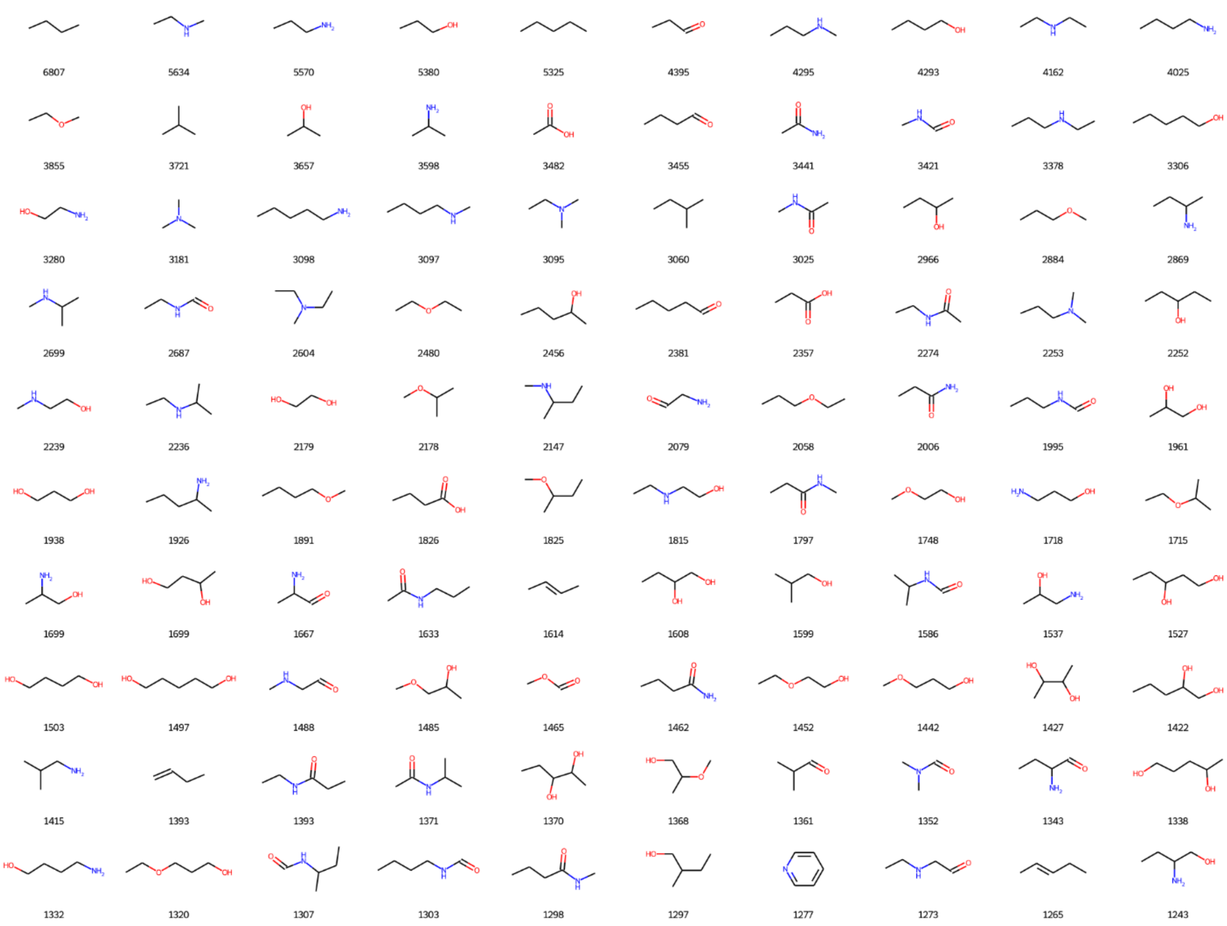

Figure S6. Top 100 most frequently occurring amine–acid enumeration products found as substructures in Drugbank, labeled by number of drugs each product is found in.

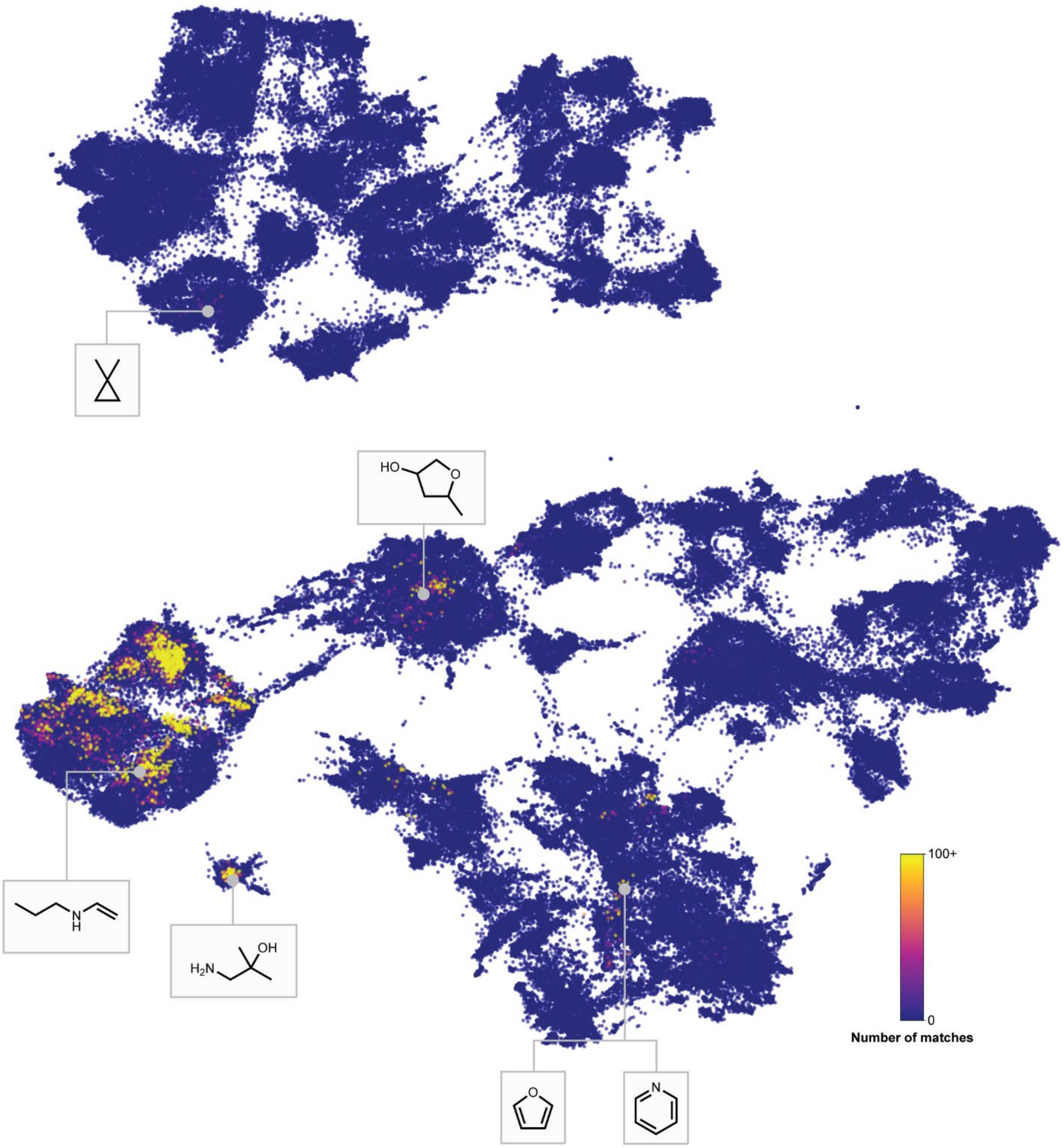

Figure S7. Uniform Manifold Approximation and Projection (UMAP) visualization of all 222,740 enumerated coupling products of a simple amine–carboxylic acid coupling pair. Products are clustered on computed 2048-bit Morgan fingerprints with radius 2, and colored on the frequency of their incidence in the Drugbank database.

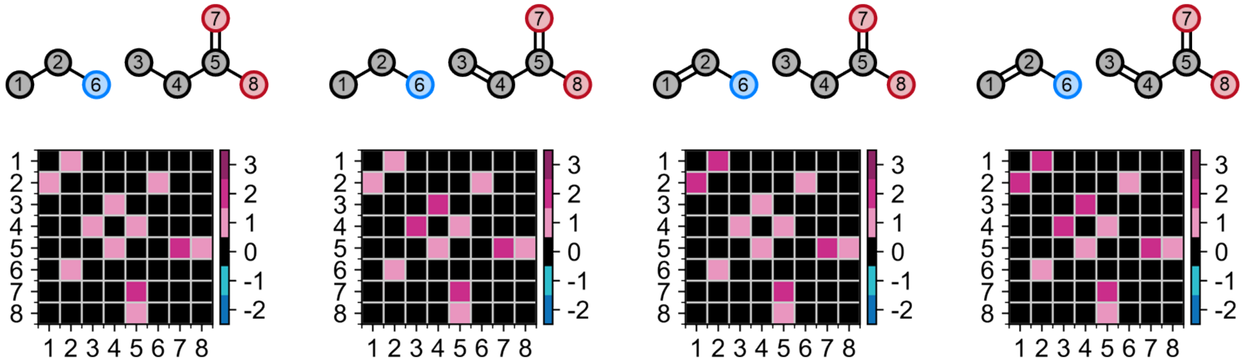

Figure S8. The four starting material hybridization permutations, and their matrix representations. From left to right: *sp*^{3} amine and *sp*^{3} acid, *sp*^{3} amine and *sp*^{2} acid, *sp*^{2} amine and *sp*^{3} acid, and *sp*^{2} amine and *sp*^{2} acid.

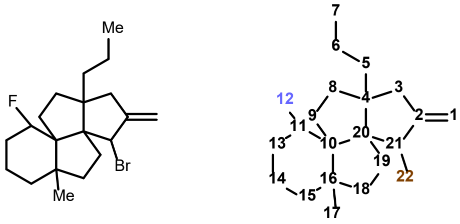

SMILES: C=C1CC2(CCC)CCC34C(F)CCCC3(C)CCC24C1Br

Figure S9. Example of a molecule with multiple fused rings and substituents, its canonical SMILES as encoded by RDKit, and the indices of its individual atoms as returned by the rdkit.Chem.GetAdjacencyMatrix() function.

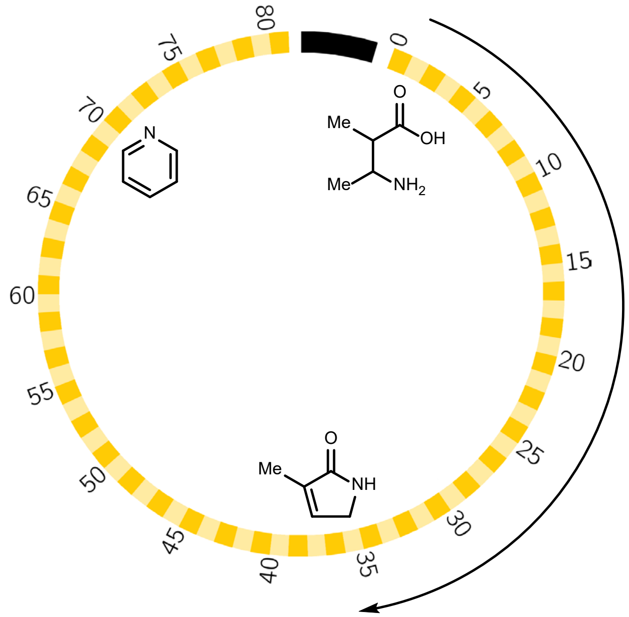

Figure S10. Examples of the distribution of enumerated amine–acid coupling products along the chord diagram. The higher the number on the perimeter of the chord diagram, the more bond edits are needed to transform a two-carbon amine and three-carbon acid into a structure placed there.

	search: **C=C**	
condition	keep aromaticity	clear aromaticity
matching bonds	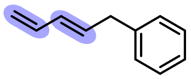	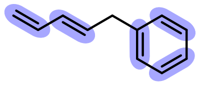
number of matches	2	5

Figure S11. Demonstration of the effects of clearing a molecules' aromatic flags in RDKit before performing substructure search. When aromatic flags are cleared, aromatic regions no longer match with alkenes.

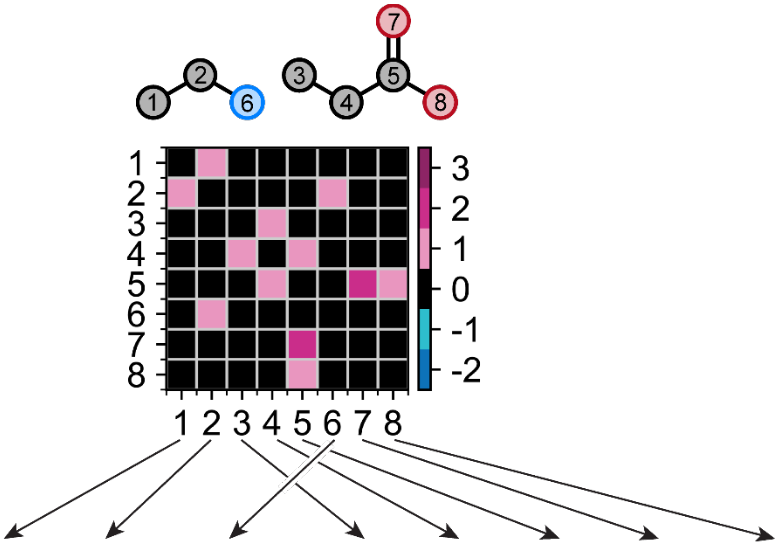

SMARTS: [CX4] [CX4] [NX3H2] . [CX4] [CX4] [CX3] (=[O]) [OX2H1]

Figure S12. Construction process of the mapper from matrix indices to the corresponding atoms' positions on the SMARTS search string.

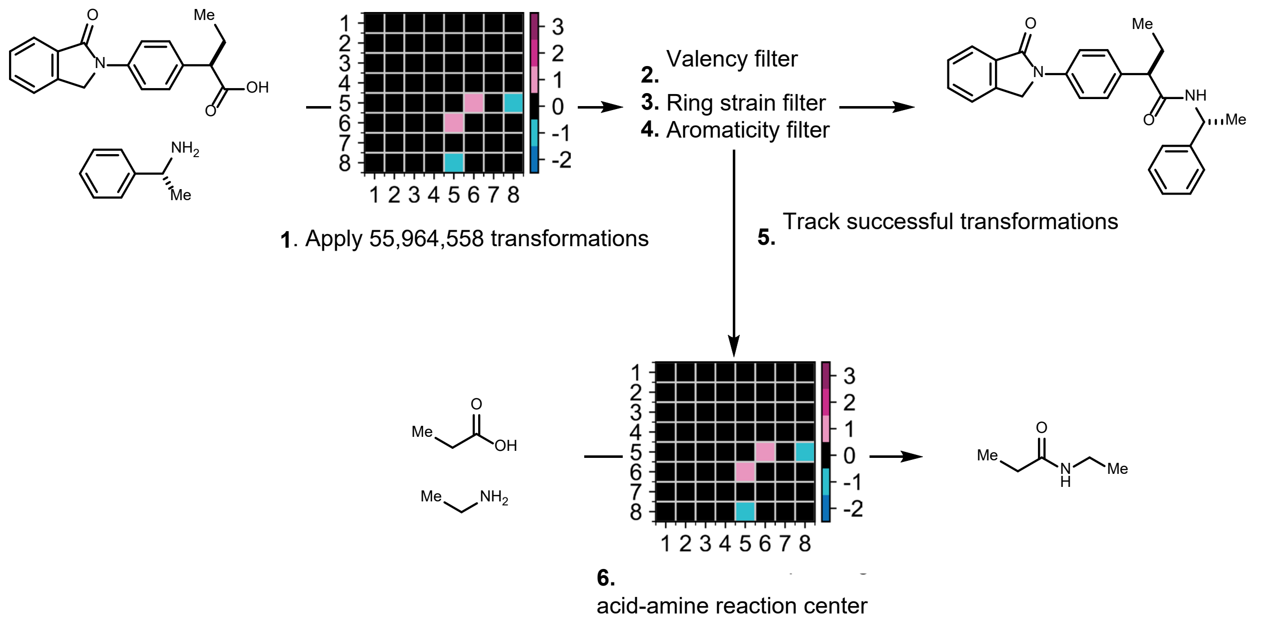

Figure S13. Flowchart of in silico late-stage diversification methodology.

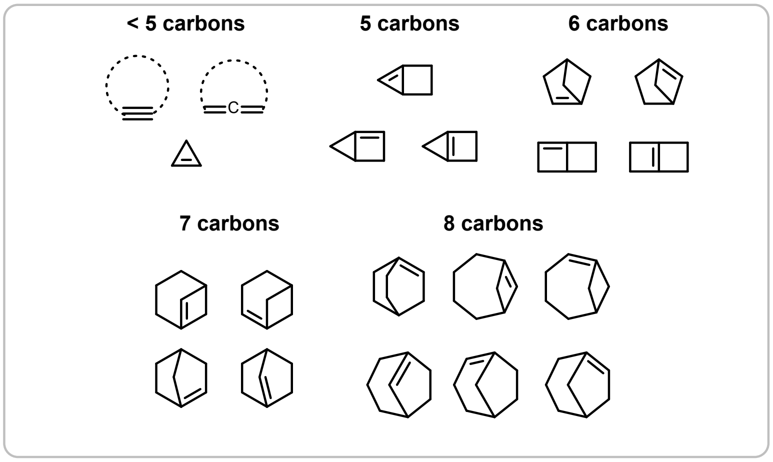

Figure S14. Structural motifs containing Anti-Bredt motifs or other sources of substantial ring strain, which are removed from the late stage-diversified set.

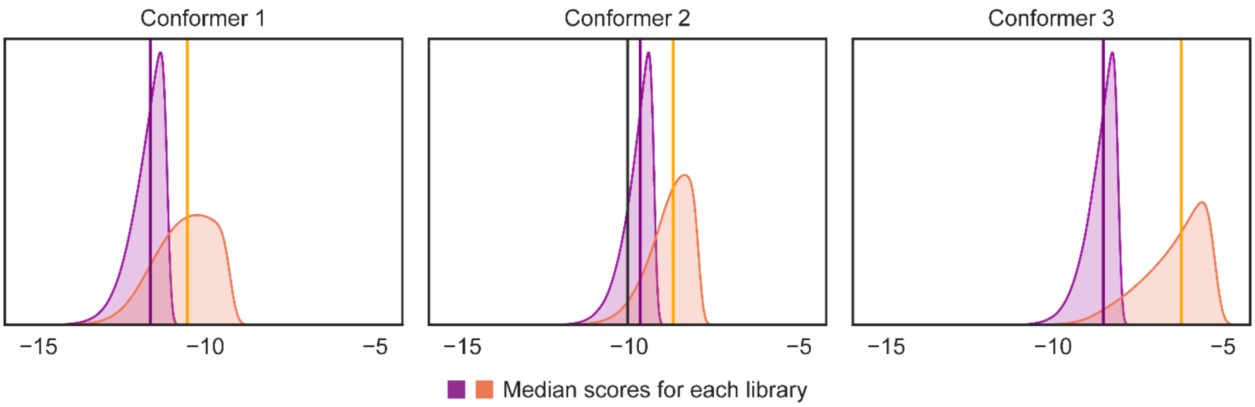

Figure S15. Docking score distributions of an M^{pro} inhibitor analog, across three M^{pro} conformers. Purple lines represent distributions and medians of a virtual library generated via amine–acid reaction enumeration, filtered to six of fewer bond edits from the building blocks, while orange lines represent that of amide coupling with diverse amines, filtered to 29 or less heavy atoms.

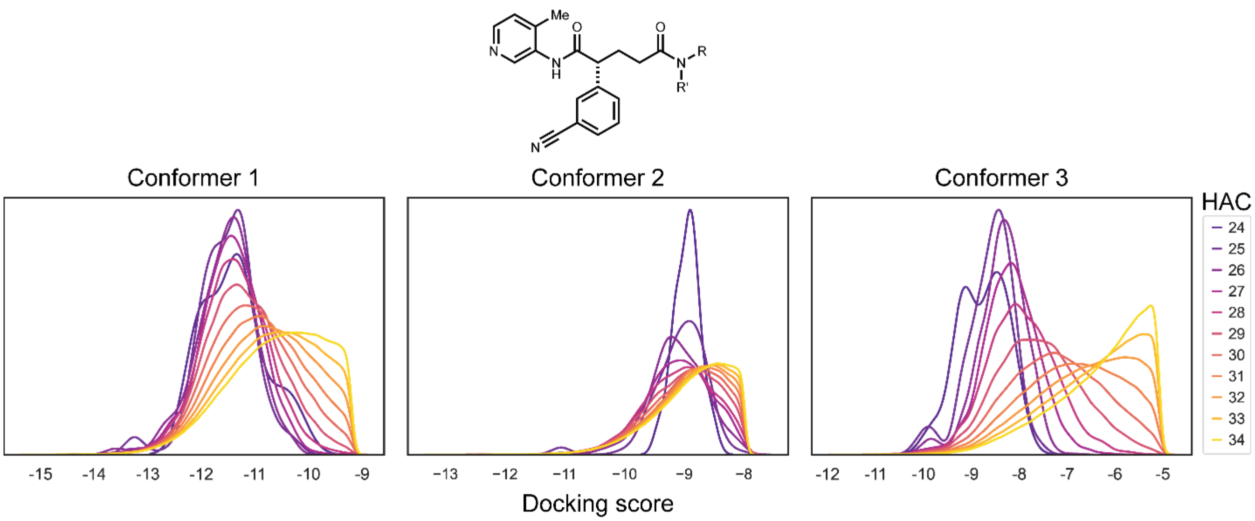

Figure S16. Trellised KDE plots of docking scores of diverse amides formed from coupling a small molecule acid with diverse amines retrieved from PubChem. Each plot includes all structures with heavy atom count (HAC) equal to or less than the indicated value.

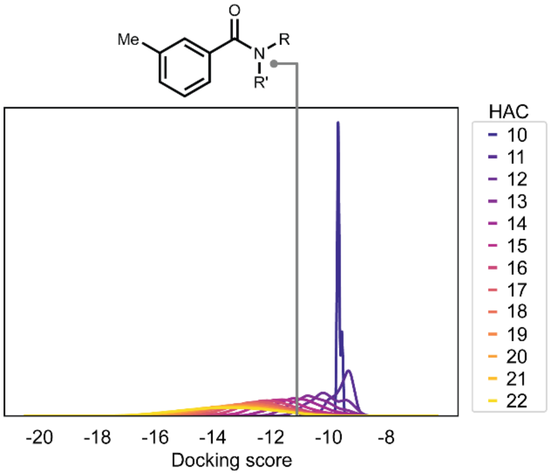

Figure S17. Trellised KDE plots of docking scores of diverse amides formed from coupling 3-methylbenzoic acid with diverse amines retrieved from PubChem.

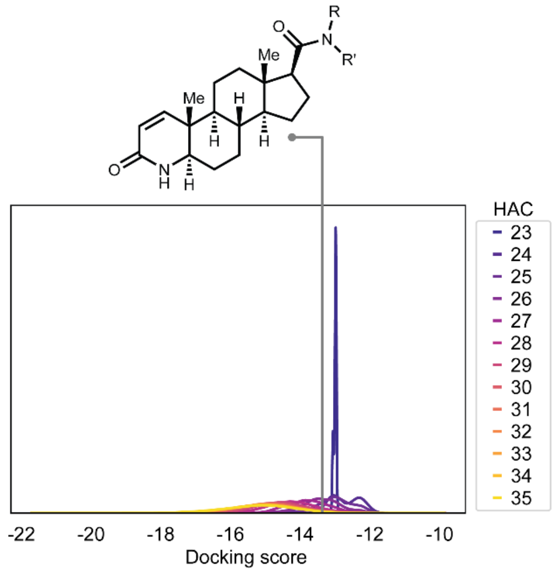

Figure S18. Trellised KDE plots of docking scores of diverse amides formed from coupling the acid building block of finasteride with diverse amines retrieved from PubChem.

Supplementary Notes

Additional notes and/or workflow for selected General Procedures

General Procedure 1

For manual encodings:

1. Initialize a blank matrix, either with np.zeros((l, l))where l is the number of atoms in the system, or l copies of a list of zeros with length l.
2. Encode a list of bond indices and orders as (m, n, b) triplets, where m and n are indices of its joining atoms, and b is its bond order. Iterate through the list, setting the (m,n) and (n,m) entries to b.

Larger molecules can be quickly encoded through RDKit's built-in rdkit.Chem.GetAdjacencyMatrix() function, setting useBO=True to encode bond orders. Otherwise, all bonds will be encoded as 1, regardless of bond order.

Except for General Procedure 4, aromatic molecules are Kekulized through the rdkit.Chem.Kekulize function, setting clearAromaticFlags=True to convert aromatic motifs into alternating single and double bonds, to avoid half bond orders appearing in matrices.

To convert an adjacency matrix into a molecule:

1. Initialize a blank Chem.RWMol()object
2. Iterate through the list of atomic numbers, performing AddAtom()to place all atoms as individual nodes.
3. Iterate through the lower triangle of the adjacency matrix, using AddBond()to place all bonds
4. Perform Chem.SanitizeMol()to check for chemically invalid structures, such as those that exceed permitted maximum valencies. This step can be switched off to manually inspect the final product for errors.

General Procedure 2

Given a single atom's maximum total bond order *t*, and maximum individual bond order *b*, a full enumeration of a single adjacency matrix row can be performed using python's itertools.product()function.

Using a single oxygen atom as example, *t* = 2 and *b* **=** 2, as a neutral oxygen can make 2 total bonds, as either 2 single bonds or 1 double bond. In a 3-atom system, its corresponding row is generated through itertools.product([0,1,2],repeat=3). This produces the following output:

(0, 0, 0)	(1, 0, 0)	(2, 0, 0)
(0, 0, 1)	(1, 0, 1)	(2, 0, 1)
(0, 0, 2)	(1, 0, 2)	(2, 0, 2)
(0, 1, 0)	(1, 1, 0)	(2, 1, 0)
(0, 1, 1)	(1, 1, 1)	(2, 1, 1)
(0, 1, 2)	(1, 1, 2)	(2, 1, 2)
(0, 2, 0)	(1, 2, 0)	(2, 2, 0)
(0, 2, 1)	(1, 2, 1)	(2, 2, 1)
(0, 2, 2)	(1, 2, 2)	(2, 2, 2)

This is the full set of 1 × 3 tuplets, where each element is an integer between 0 and 2 (in general cases, between 0 and *b*). However, most of these products exceed the octet rule, as oxygen cannot make more than 2 bonds in total. To filter out the invalid results, all tuples are individually summed, and only the ones that sum to *t* or below are kept.

General Procedure 3

1. Create and store starting material matrices (Fig. S7)
2. Iterate through product matrices generated in General Procedure 2:
    i. Convert matrix to mol object following General Procedure 1
    ii. Convert mol objects to SMILES
    iii. Generate (and save, if desired) transformation matrices from each starting material by subtracting starting material matrices from that of the product
    iv. For each transformation matrix, compute graph edit distance by taking the absolute value of the transformation matrix, summing all its elements, then dividing by 2.
    v. Compile all data in a spreadsheet, where each row begins with a product SMILES string, and is followed by its bond edit distances from each variation of starting material.
3. For compiling possible graph edit distances for each individual product (i.e. the set of possible single molecules from the set of product molecular systems), initialize a blank defaultdict(list). This behaves similar to a regular python dictionary, except the value for each key is initialized to an empty list, and can be appended to in-place.
4. Iterate through the spreadsheets produced in step 2:
    i. Split each SMILES string into that of individual molecules, keeping only those which contain 4 or more heavy atoms.
    ii. For each SMILES string passing step (i), locate its minimum graph edit distance among all possible starting materials. Append this distance to the corresponding dictionary value, with the SMILES string as key.

5. Canonize the SMILES keys in the dictionary generated in step 4, and further re-compile graph edit distances, this time corresponding to each product's canonical SMILES. This step can theoretically be executed during step 4 (i) to achieve the same result, but is delayed to avoid work duplication.
6. Iterate through the dictionary created in step 5, only keeping the minimum graph edit distance for each product SMILES string. Compile and save SMILES, minimum distance, and number of heavy atoms (computed during step 4i) into a spreadsheet.

General Procedure 4

1. Load Drugbank structures to be searched within.
2. Load the spreadsheet produced in step 6 of General Procedure 3, iterating through the column containing structure SMILES:
    i. Initialize running total of matches to 0
    ii. Run Chem.SanitizeMol() to convert bonds to aromatic, if applicable.
    iii. Search for presence of structure in each Drugbank structure. If a match is found, add 1 to the running total
3. Collect total number of matches into a column, append to the right of the spreadsheet loaded in step 2, and save.

For producing circos input files:

4. Load the single drug molecule to be searched against.
5. Sort the spreadsheet of SMILES by minimum bond edit distance.
6. Going down the column, search each substructure SMILES within the single drug molecule, this time collecting the number of matches by each individual substructure (compared to only the presence or absence in step 2 (iii)).
7. Append the column produced in step 6 to the spreadsheet
8. Sort the spreadsheet by increasing number of matches. This ensures that, when the chord diagram is plotted, the lines showing highest incidence of matches are plotted on top. In Pandas, the index of each row is preserved on sorting, so the position of each substructure on the chord diagram's outer circle is not lost when sorted by matches instead of graph edit distance.
9. Iterate through spreadsheet rows to write the linker text file in the following format:

   [name of source band] [source band position (2 values)] [name of target band] [target band position (2 values, determined by row index)] [colors]

   Details about the formatting of circos links can be found at: http://circos.ca/documentation/tutorials/links/basic_links/.

General Procedure 5

1. Iterate through product SMILES strings:
    i. Convert into mol object and sanitize.
    ii. Add explicit hydrogens – this can appreciably slow down the next step. Skipping this step will produce similar results, albeit compromising on accuracy.
    iii. Embed molecule in 3D and calculate NPR1 and NPR2.

General Procedure 6

1. Visualize the molecule(s) on which the transformation is to be performed.
2. Construct SMARTS strings to uniquely match with the desired functional groups. This can be done in tandem with step 1, where additional atoms can be attached to the substrate, or important atoms modified in-place to identify them for transformation. These atoms should be defined such that they can be effectively removed at the end of a transformation. For example, if the substrate already contains C-F bonds, then fluorine atoms should be avoided as markers.
3. Based on the construction of SMARTS strings and atom indices on the product matrices, construct a method to map each atom index onto the order in which it appears in the SMARTS string (Figure S18)
4. Load the substrates as Chem.RWMol()objects.
5. Load the relevant transformation matrices corresponding to the hybridization of the amine and acid groups on the substrate(s). If these were saved during General Procedure 3, step 2 (iii), they can be loaded directly. Otherwise, these can be computed on-the-fly by loading the product matrices computed in General Procedure 2, and subtracting from them the relevant starting system (Fig. S8).
6. Iterate through the transformation matrices:
    i. For each non-zero entry in the lower triangle (indicating a change in bond order), use the mapping constructed in step 3 to determine the indices of its joining atoms within the substrate.
    ii. Obtain the current bond order between the indicated atoms
    iii. Compute the new bond order by adding the bond order change to the current bond order, and add the bond between the computed atom indices
    iv. Check the product for violations of any valency rules. If none are found, return the product system.
7. Iterate through all valid products returned by step 6, checking for the presence of a coupling product, that is, a product containing at least one atom from the acid group, and one from the amine group. This can be done either by searching for functional groups on the relevant atoms, or labeling the groups themselves as isotopes in step 1.
8. Iterate through products that passed the coupling check in step 7, filtering out those with significant ring strain.
9. Remove any isotope and/or functional group tagging introduced in step 1.
10. Remove duplicate structures, keeping only the copy with the smallest graph edit distance from the substrates.
11. Perform further filtering as desired. This can include filtering out *sp*^{2} systems that had their aromatic ring broken, or further searching for undesired structural motifs.
12. Save the resultant data, keeping at least the cleaned SMILES and the index of the product/transformation matrix that produced it.

General Procedure 7

1. Load the appropriate system of a 2-carbon amine and 3-carbon acid, with hybridizations corresponding to that of the larger druglike substrate system, and modifying atoms for tracking as desired (cf. step 1 of General Procedure 7)
2. Repeat steps 4-9 of General Procedure 7 on the simple system, but only using the transformation matrices whose indices were returned by step 11.
3. The resultant dataset has each valid transformation matrix mapping to two products – the functionalization of the druglike substrate, and the corresponding reaction center. These products can be further used to compute pairs of molecular properties and shapes.

Experimental details on the synthesis of 51, 52, and 53

To a solution of 2,4,6-triphenylpyrylium tetrafluoroborate (163.3 mg, 0.412 mmol, 1.0 equiv.) in anhydrous ethanol (0.412 mL) was added benzylamine **49** (47.3 µL, 0.433 mmol, 1.05 equiv.) at room temperature. The solution was then stirred at 80 °C for 4 h. After reaction completion, the reaction solution was cooled to room temperature, then dropwise transferred into 3 mL of diethyl ether to precipitate the pyridinium salt. After stirring the mixture for 10 minutes, the solid was subjected to vacuum filtration and washing with diethyl ether to retrieve N-benzyl-2,4,6-triphenylpyridinium tetrafluoroborate **52**, to be directly used without purification.

A separate vial was prepared with benzoic acid **50** (50.3 mg, 0.412 mmol, 1.0 equiv.) and thionyl chloride (45.1 µL, 0.412 mmol, 1.0 equiv.) in dichloromethane (4.12 mL). The vial was stirred at 40 °C for 2 h, then solvent was removed *in vacuo*. To the crude benzoyl chloride, anhydrous THF (4.12 mL) was added, and the vial cooled to -78 °C in a dry ice-acetone bath. Over 10 minutes, a 1M solution of lithium tri-tert-butoxyaluminum hydride in THF (412 µL, 0.412 mmol, 1.0 equiv.) was added dropwise. The reaction vial was continuously stirred at -78 °C for 2 h, then quenched with 1M HCl (1 mL) and warmed to room temperature. The reaction mixture was diluted with water (10 mL). Extraction in triplicate with DCM (3 x 10 mL), drying of the combined organic layers with Na_2SO_4, and removal of solvent in vacuo yielded crude benzaldehyde **53** that was directly used in later reactions.

The crude benzaldehyde residue was taken up in anhydrous DMSO (2.06 mL) and the resulting solution was transferred to a 2-dram vial containing benzyl pyridinium salt **52** and solid cesium carbonate (134.3 mg, 0.618 mmol, 1.5 equiv.). The reaction was stirred at 60 °C for 16 h, then the mixture was diluted with water (30.0 mL) and extracted with DCM (3 x 10 mL). The combined organic layers were dried over anhydrous Na_2SO_4, filtered, and concentrated in vacuo. To the crude residue, solid indium (III) bromide (73.1 mg, 0.412 mmol, 1.0 equiv.) was added along with chloroform (4.12 mL). The reaction mixture was heated to 60 °C for 16 h. After dilution with brine (10.0 mL) and extraction with DCM (3 x 5.0 mL), the combined organic layers were dried over anhydrous Na_2SO_4, filtered, and concentrated in vacuo.

This crude mixture was dissolved in anhydrous ethanol (4.12 mL) and solid portions of $NaBH_4$ (15.6 mg, 0.412 mmol, 1.0 equiv.) were added at room temperature. The solution was stirred at room temperature for 2 hours, cooled to 0 °C, and quenched with aqueous acetic acid (1.0 M, 2 mL) and water (10 mL). Extraction with DCM (3 x 5 mL), drying of the combined organic layers with Na_2SO_4, and removal of solvent in vacuo yielded the corresponding reduced alcohol product. Purification by flash chromatography (silica gel, eluent: 10-20% EtOAc/hexanes) afford 2,2-diphenylethanol **51** (36.9 mg, 45% yield) as a white solid. Proton and Carbon NMR for this compound is displayed as follows:

1H NMR (500 MHz, $CDCl_3$) ∂ 7.36-7.33 (m, 4H), 7.31–7.22 (m, 6H), 4.25-4.22 (m, 1H), 4.20 – 4.18 (m, 2H). 13C NMR (125 MHz, CDCl3) ∂ 141.37, 128.74, 128.32, 128.32, 126.84, 66.16, 53.66. HRMS (ESI): calculated $C_{14}H_{15}O^+$ $[M + H]^+$: 199.1117, found: 199.1124.

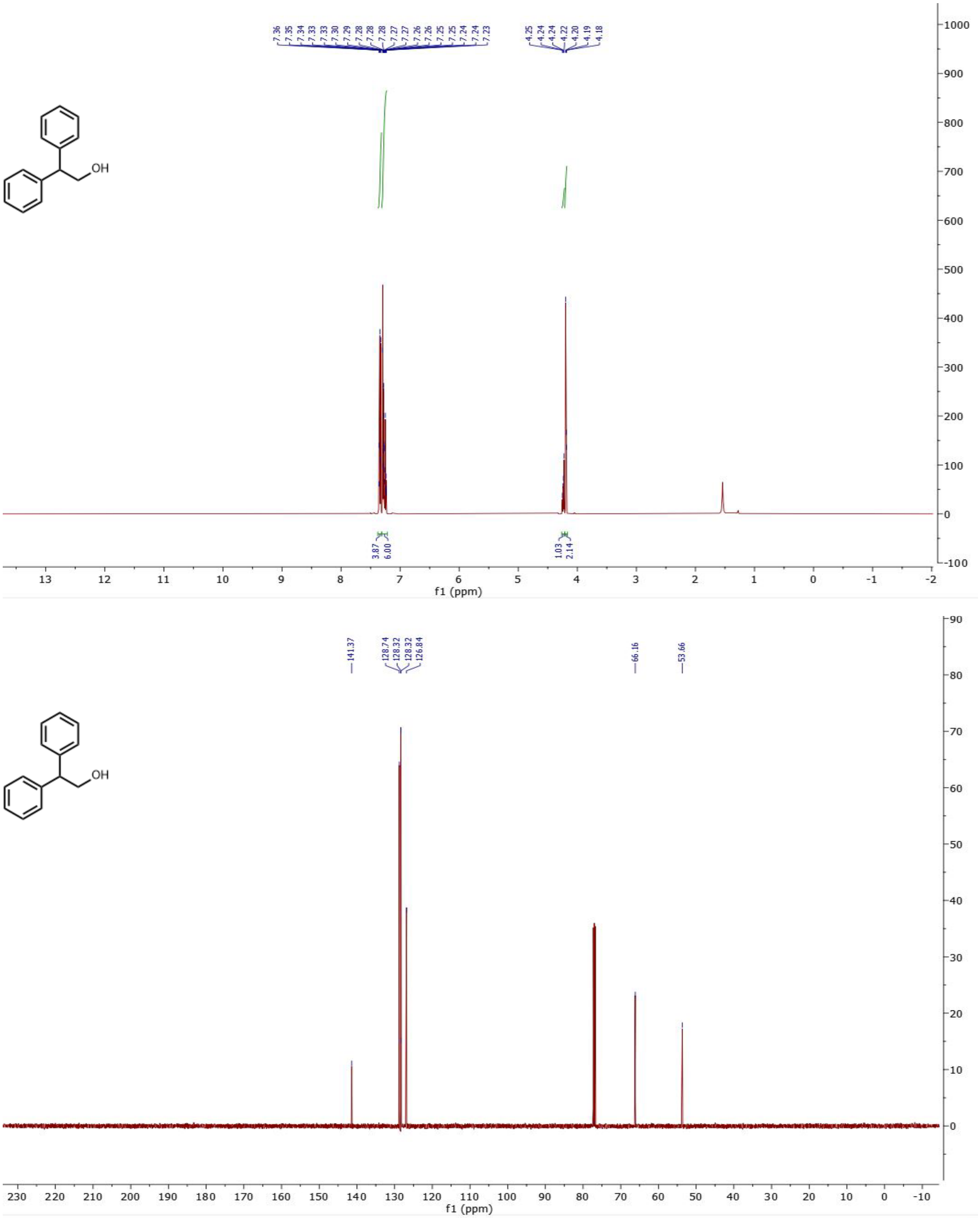

Figure S19. 1H and 13C NMR spectra of 2,2'-diphenylethanol (**51**).
